# *RFXANK*: A Novel Immune-Related Biomarker for Hepatocellular Carcinoma

**DOI:** 10.3390/genes17040406

**Published:** 2026-03-31

**Authors:** Taimei Qu, Lv Tian

**Affiliations:** 1 Department of Ultrasound, The Second Medical Center, Chinese PLA General Hospital, Beijing 611731, China; 2 Institute of Fundamental and Frontier Sciences, University of Electronic Science and Technology of China, Chengdu 610054, China

**Keywords:** hepatocellular carcinoma, *RFXANK*, immune checkpoints, biomarkers, molecular mechanism, RAF1

## Abstract

**Background**: Hepatocellular carcinoma (HCC) represents an extremely lethal malignancy on a global scale. The clinical significance and molecular mechanisms of the immune-related gene *RFXANK* in HCC remain unclear. This study seeks to elucidate the clinical implications and diagnostic utility of *RFXANK* in HCC, while further exploring its underlying molecular mechanisms. **Methods:** Expression differences of *RFXANK* in pan-cancer and HCC were analyzed using the TCGA and GEO (GSE45267) databases. Its diagnostic efficacy was evaluated by Cox regression, Kaplan–Meier survival curves, and ROC curves. Potential functional pathways were explored through GO, KEGG, and GSEA enrichment analyses. The correlation between *RFXANK* and immune cell infiltration, as well as immune checkpoint molecules, was analyzed using the ssGSEA algorithm and CIBERSORTx. In vitro, siRNA interference was employed to knock down *RFXANK* expression in Huh-7 and MHCC97H cells. The effects on cell proliferation and RAF1 protein levels were assessed using a CCK-8 assay and Western blot, respectively. **Results**: *RFXANK* was significantly overexpressed in HCC tissues and was closely associated with aggressive clinical features, including pathological T stage, histological grade, and AFP levels. Multivariate Cox regression analysis confirmed that *RFXANK* was an independent risk factor for survival in HCC patients (HR = 1.871). The area under the ROC curve (AUC) was 0.939, demonstrating excellent diagnostic predictive value. Enrichment analysis revealed a significant association with the cell cycle, PPAR signaling pathway, and lipid metabolism pathways. Immune infiltration analysis further revealed that *RFXANK* expression was significantly positively correlated with Th2 and TFH cells, as well as key immune checkpoint molecules such as PD-1, CTLA4, and LAG3, suggesting distinct features of immune polarization and an inhibitory microenvironment. In vitro cellular experiments demonstrated that knocking down *RFXANK* significantly inhibited the proliferative capacity of HCC cells and reduced RAF1 protein expression. **Conclusions**: *RFXANK* may promote HCC progression by driving a multidimensional proliferation–metabolism–immunity mechanism. *RFXANK* holds promise as a novel biomarker for diagnostic assessment and a potential therapeutic target for HCC patients.

## 1. Introduction

Causing approximately 750,000 deaths annually, hepatocellular carcinoma (HCC) accounts for the majority of primary liver cancer cases worldwide—about 75%—and continues to be associated with a poor prognosis [1,2]. Notwithstanding refinements in therapeutic strategies such as hepatic resection, transplantation, and ablative techniques, coupled with breakthroughs in molecularly targeted drugs and immune checkpoint inhibitors, the long-term prognosis for HCC remains suboptimal, with 5-year survival rates failing to meet clinical expectations [2,3]. This is primarily attributed to the high heterogeneity of HCC, its insidious onset, and the high postoperative recurrence rate [1]. Furthermore, although existing clinical staging systems (e.g., BCLC and TNM) can assist in clinical decision-making, they still have limitations in assessing individualized prognosis. Currently, clinically utilized biomarkers for HCC, such as alpha-fetoprotein and glypican-3, exhibit insufficient sensitivity and specificity to meet the demands of precision diagnosis and treatment [4]. Although existing serum markers (e.g., AFP, AFP-L3, DCP) have some clinical value, the association between molecular characteristics and these markers remains unclear. Furthermore, there is a lack of highly sensitive and specific predictive tools for prognosis assessment, minimal residual disease monitoring, and treatment response prediction [5]. While immune checkpoint blockers targeting PD-1/PD-L1 and CTLA-4 have demonstrated significant efficacy in some patients, treatment responses show substantial individual variability, and the overall response rate remains low [6]. The immunosuppressive state, along with spatial and temporal heterogeneity within the tumor microenvironment, further limits the broad application of current immunotherapies [7]. Consequently, there is an urgent need to discover novel molecular markers (such as long non-coding RNAs, RNA modification-related molecules, and immune microenvironment signature genes) and immune regulation targets (e.g., SLAMF7, MASP1, KIF20A, CD177^+^ Tregs) to optimize patient stratification, overcome immunotherapy resistance, and enhance the precision of combination therapy strategies [8,9].

In recent years, the landscape of cancer research has evolved from a tumor-centric genetic perspective to a multidimensional exploration of the complex interplay within the tumor microenvironment [10,11]. As a specialized immune organ, the liver harbors a complex cellular composition (e.g., infiltrating immune cells) that plays a “double-edged sword” role in the development and progression of HCC [12]. Tumor cells can establish a microenvironment conducive to tumor growth and immune evasion by downregulating immune recognition molecules, recruiting immunosuppressive cells (e.g., Tregs, MDSCs), and activating immune checkpoint pathways [12,13,14]. Against the backdrop of rapid advances in bioinformatics, identifying key factors involved in gene transcriptional regulation, immune recognition, and signal transduction has become a crucial approach to untangle the malignant phenotype of tumors and their interplay with the host immune system, thereby facilitating the exploration of new strategies for the precise management of HCC [14,15].

Regulatory factor X-associated ankyrin-containing protein (*RFXANK*) plays a critical role in the transcriptional regulation of major histocompatibility complex class II molecules. Through its internal ankyrin repeats, this protein interacts with RFX5 and RFXAP to form a trimeric complex [16]. This complex specifically binds to the X-box motif in the promoters of MHC class II genes, subsequently recruiting the MHC class II transactivator to initiate the transcription and expression of MHC class II molecules [16]. Such a mechanism ensures the robust surface presentation of MHC class II molecules, a prerequisite for effective immune recognition by antigen-presenting cells. It serves as an essential prerequisite for antigen recognition by CD4^+^ T helper cells and the initiation of adaptive immune responses [17]. Clinical studies have confirmed that loss-of-function mutations in the *RFXANK* gene are the primary cause of Bare Lymphocyte Syndrome (BLS) complementation group B [18]. In the field of oncology, previous studies have established that members of the RFX family (RFX1–8) are closely associated with tumorigenesis and progression: RFX1 is aberrantly expressed in various malignancies and can interfere with multiple cellular physiological processes, making it a promising therapeutic target in cancer treatment [19]; Evidence from transcriptomic datasets underscores a robust association between RFX2 dysregulation and the pathological evolution of ovarian cancer and non-small cell lung cancer [20]; RFX3 has been identified as a driver of breast cancer development [21]; RFX6 participates in the pathological progression of liver cancer by regulating tumor invasiveness and T cell-mediated immune responses [22]; RFX7 mutations have been confirmed as cancer drivers in chronic lymphocytic leukemia [23]. In mouse models, loss of RFX7 accelerates the development of B-cell lymphoma [24]. RFX7 expression levels are closely correlated with tumor cell differentiation and favorable patient prognosis. Furthermore, proteomic and interactomic studies suggest that the interaction profile between *RFXANK* and caspase-2 indicates non-apoptotic functions of *RFXANK* in regulating MHC class II gene expression [25]. It has been demonstrated that *RFXANK* can cooperate with ANKRA2 and RFX7 to participate in p53-mediated transcriptional programs, with p53 serving as a key tumor suppressor within cells [26]. In summary, *RFXANK* appears to harbor significant, albeit elusive, biological significance in malignant transformation. Therefore, elucidating the expression landscape of *RFXANK* in HCC and its role in re-engineering the immune landscape will provide critical insights into HCC immune-escape mechanisms. Furthermore, these insights hold substantial potential for translating *RFXANK* into a novel diagnostic tool and a sensitizing target for combinatorial immunotherapy.

Although the fundamental biological functions of *RFXANK* have been preliminarily explored, its expression pattern, clinical diagnostic value, and specific immunoregulatory mechanisms in HCC remain unclear. Preliminary analyses based on TCGA and the GEO database suggest that *RFXANK* exhibits significantly heterogeneous expression in HCC tissues, and its expression level correlates well with the overall survival (OS) of patients. Based on these findings, this study aims to explore the molecular mechanisms of *RFXANK* in HCC progression, provide novel molecular evidence for the precise diagnostic assessment of liver cancer, and identify potential intervention targets.

## 2. Materials and Methods

### 2.1. Data Sources and Preprocessing

Pan-cancer RNA-seq expression profiles of *RFXANK* were retrieved from UCSC Xena (https://xenabrowser.net/datapages/, accessed on 25 November 2025) in TPM format. These TCGA datasets had been uniformly recomputed via the Toil process [27]. For the TCGA-LIHC project, expression data for unpaired samples were sourced from the GDC portal (https://portal.gdc.cancer.gov/analysis_page?app=Downloads, accessed on 25 November 2025) in Level 3 HTSeq-FPKM format. To ensure comparability across samples, FPKM estimates were recalibrated as TPM values and underwent log2-scaling prior to all downstream computational assessments. Regarding the GSE45267 dataset [28], raw data were accessed from the GEO (https://www.ncbi.nlm.nih.gov/geo/download/?acc=GSE45267, accessed on 25 November 2025) using the GEOquery package (v2.54.1) [29]. During preprocessing, probes mapping to multiple genes were filtered out; for gene symbols represented by several probes, only the highest-intensity signal was selected for further analysis. Systematic variations across microarrays were corrected via the normalizeBetweenArrays algorithm from the limma package (v3.42.2) [30]. The entire statistical pipeline and data visualization were conducted using R software (v4.2.1).

### 2.2. Differential Expression Analysis of RFXANK

Inter-group differences in *RFXANK* expression across various cancers were assessed using the Mann–Whitney U algorithm. Prior to the comparative analysis, the Shapiro–Wilk test was performed to assess the distribution of the GSE45267 dataset and its associated sample. For cohorts meeting normality assumptions, an independent-samples *t*-test was used; otherwise, the Mann–Whitney U test was prioritized for the GSE45267 dataset. All visualizations were implemented via ggplot2 (v3.3.3), maintaining a significance cutoff of 0.05.

### 2.3. Differential Gene Expression Analysis and Correlation Analysis of RFXANK

Differential expression analysis (DEA) was performed on Level 3 HTSeq-Counts from the TCGA-LIHC cohort utilizing the DESeq2 (v1.36.0) package [31]. Using the R stats package (v3.6.3), we evaluated the correlation between *RFXANK* and other genes from TPM-normalized data. To visualize the DEA results, volcano plots were constructed using a significance threshold of |log2(FC)| > 0.9 and p.adj < 0.05. Subsequently, the top 10 genes most strongly associated with *RFXANK* were identified by ranking the absolute Pearson correlation coefficients in descending order. These candidates were then used to generate a co-expression heatmap. All visualizations, including volcano plots and heatmaps, were implemented via the ggplot2 package (v3.3.3).

### 2.4. Clinical Correlation Analysis and Survival Prognosis of RFXANK Expression

Prognostic significance was evaluated using survival data derived from a previously published cell study [32]. Using the median *RFXANK* expression level across the entire study cohort as the cutoff value, patients were divided into two groups: those with expression levels above the median were classified as the *RFXANK* high-expression group, whilst those with expression levels at or below the median were classified as the *RFXANK* low-expression group [33]. Kaplan–Meier survival curves for the LIHC cohort were modeled to estimate overall survival (OS) probabilities, with graphical plots rendered via the survminer package (v0.4.9). Beyond whole-cohort analysis, we performed clinicopathological stratification, accounting for variables such as age, sex, and body mass, to uncover latent survival heterogeneity. The association between these clinical features and *RFXANK* expression was further quantified and visualized using ggplot2 (v3.3.3). To determine the diagnostic efficacy of *RFXANK* as a diagnostic indicator, Receiver Operating Characteristic (ROC) curves were constructed utilizing the pROC library (v1.17.0.1). Independent predictors of survival were ultimately identified through stepwise univariate and multivariate Cox proportional hazards modeling, with *RFXANK* expression bifurcated by its median value. These regression outcomes were summarized through forest plots.

### 2.5. Functional Enrichment Analysis of RFXANK in LIHC

To explore biological functions and signaling pathways, we employed the R package clusterProfiler (v4.4.4) for performing comprehensive enrichment evaluations, specifically covering GO, KEGG, and GSEA [34]. The org.Hs.eg.db (v3.10.0) annotation database was utilized to ensure seamless mapping and transformation of gene nomenclature. To quantify the association between *RFXANK* and the identified biological pathways, Z-scores were computed via the GOplot package (v1.0.2) [35]. The GSEA was benchmarked against the “c2.cp.all.v2022.1.Hs.symbols.gmt” collection [36], which encompasses all canonical pathway definitions. Statistical significance for enriched terms was strictly defined by a threshold of FDR < 0.25 alongside an adjusted *p*-value below 0.05. Finally, all resulting data were visualized using ggplot2 (v3.3.3).

### 2.6. Immunoinfiltration Analysis of RFXANK

Based on the ssGSEA algorithm from the R package GSVA (version 1.46.0) [37], the correlation between *RFXANK*, its top 10 positively and negatively correlated genes, and 24 immune cells was calculated using the 24 immune cell markers provided by the journal Immunity [38]. All *p*-values were adjusted using the Benjamini–Hochberg method for FDR, and a corrected q-value of <0.05 was considered statistically significant. The infiltration proportions of 22 immune cell types in LIHC samples were calculated using the corresponding markers available on the CIBERSORTx website (https://cibersortx.stanford.edu/, accessed on 25 November 2025). The potential association between *RFXANK* expression and immune checkpoints (specifically TNFRSF4, PDCD1, TNFRSF18, CTLA4, LAG3, and TIGIT) was quantified using the Spearman correlation analysis. All the aforementioned statistical analyses and visualizations were performed using the circlize package (version 0.4.12).

### 2.7. Protein–Protein Interaction Analysis

To identify potential protein–protein interaction (PPI) networks associated with RFXANK, the GeneMANIA online tool (https://genemania.org/search/homo-sapiens, accessed on 25 November 2025) was employed to predict its interacting proteins.

### 2.8. Cell Lines and Culture

Huh7 and MHCC97H cell lines (Zhong Qiao Xin Zhou Biotechnology Co., Ltd., Shanghai, China), representing human HCC, were cultured using high-glucose DMEM (Sigma, St. Louis, MO, USA) enriched with 10% FBS (ExCell Bio, Shanghai, China) and 1% penicillin–streptomycin (Solarbio, Beijing, China). A standardized incubation environment was established at 37 °C, maintaining a saturated humidity level and a 5% CO_2_ concentration for all cellular experiments.

### 2.9. Cell Transfection

*RFXANK*-specific siRNAs were generated by Sangon Biotech (Shanghai, China). The oligonucleotide sequences included: NC (Sense: 5′-UUC UCC GAA CGU GUC ACG UTT-3′; Antisense: 5′-ACG UGA CAC GUU CGG AGA ATT-3′), siRNA-1 (Sense: 5′-GUG GAC AUC AAC AUC UAU GAU TT-3′; Antisense: 5′-AUC AUA GAU GUU GAU GUC CAC TT-3′), and siRNA-2 (Sense: 5′-AGG UGA CAA CCU CGU CAC AAA TT-3′; Antisense: 5′-UUG UUG ACG AGG UGU CAC CU TT-3′). Cellular transfections were carried out via the jetPRIME transfection system (#150–15, Polyplus, Strasbourg, France), strictly following the optimized protocol provided by the manufacturer.

### 2.10. Real Time-PCR

We extracted total RNA via the standard TRIzol–chloroform protocol. In brief, following a PBS wash, cells were sequentially treated with TRIzol reagent and chloroform. Subsequent to vigorous homogenization, the resulting lysate underwent 10 min of incubation at ambient temperature, followed by centrifugation (12,000 rpm, 15 min) maintained at 4 °C. The upper aqueous layer was carefully recovered into a fresh sterile tube, where an equivalent volume of isopropanol was introduced to facilitate RNA precipitation. The obtained RNA pellet was purified using 75% ethanol, briefly air-dried, and then solubilized in RNase-free water for subsequent spectrophotometric quantification. cDNA synthesis was performed using a reverse transcription kit (TransGen Biotech, Beijing, China), adhering to the manufacturer’s protocol. Target gene transcripts were quantified via SYBR Green-based real-time PCR, employing the thermal cycling parameters detailed below: an introductory 30 s denaturation step at 94 °C, succeeded by 40 amplification cycles (94 °C for 5 s and 60 °C for 30 s). Target gene expression was normalized using the 2^−ΔΔCt^ method. Primers, synthesized by Sangon Biotech (Shanghai, China), were as follows: β-actin, F: 5′-CCTGGCACCCAGCACAAT-3′, R: 5′-GGGCCGGACTCGTCATAC-3′; *RFXANK*, F: 5′-GAGAGATTGAGACCGTTCGCT-3′, R: 5′-CAGTGGCGTCCCTCCATTC-3′.

### 2.11. Cell Proliferation Assay

Cell proliferation was assessed using the Cell Counting Kit-8 (B34304, Selleckchem, Houston, TX, USA). In brief, cells were inoculated into 96-well microplates at an initial density of 4 × 10^3^ cells per well, followed by incubation under optimal growth environments. For four successive days, a 10 μL volume of CCK-8 solution was introduced into each well at 24 h periodic intervals. After an additional 2 h of incubation, the absorbance at 450 nm was measured using a microplate reader.

### 2.12. Western Blotting

Total cellular protein was extracted using RIPA lysis buffer (Meilunbio, Dalian, China), with physical disruption achieved through a specialized sample homogenization system (M.P. Biomedicals, Santa Ana, CA, USA). Following a 10 min clarified centrifugation at 13,000× *g*, the protein-rich supernatant was harvested and denatured by blending with 4× SDS-PAGE sample loading buffer. Equal amounts of protein samples were separated by 12% SDS-PAGE (Lablead, Beijing, China) before being electro-transferred to 0.45 μm pore-size PVDF membranes. Following transfer, membranes were trimmed based on molecular weight markers and blocked with 5% non-fat milk for 1 h at room temperature. The blots were then probed with primary antibodies at 4 °C for an overnight duration, followed by secondary labeling with HRP-conjugated IgG for 1 h at room temperature. Protein bands were developed using an enhanced chemiluminescence (ECL) substrate and subsequently digitized via the E-photo imaging platform (Genscript, Piscataway, NJ, USA). Antibodies used included: *RFXANK* (Abcam, Cambridge, UK, ab236408), RAF1 (Abcam, ab137435), β-actin (Abclonal, Stamford, CA, USA, AC026), and HRP-conjugated Goat Anti-Rabbit IgG (Proteintech, Rosemont, IL, USA, SA00001).

### 2.13. Statistical Analysis

Quantitative results are presented as mean ± standard deviation (SD). We employed Student’s *t*-test to assess the differential expression of *RFXANK* when comparing LIHC tumor samples with their corresponding paracancerous normal tissues. For multi-group comparisons, one-way analysis of variance (ANOVA) was utilized. Additionally, the relationship between clinical features and *RFXANK* expression levels was examined through the non-parametric Mann–Whitney U test. The co-expression patterns of *RFXANK* with other genomic targets were explored by calculating Spearman correlation coefficients. All results were from three independent experiments. Statistical comparisons between groups in in vitro experiments were performed using Student’s *t*-test (two-tailed) and ANOVA. Statistical analyses and visualization were conducted using GraphPad Prism 8, with statistical significance defined by *p*-value < 0.05.

## 3. Results

### 3.1. Screening and Identification of Target Genes

**Figure 1:**
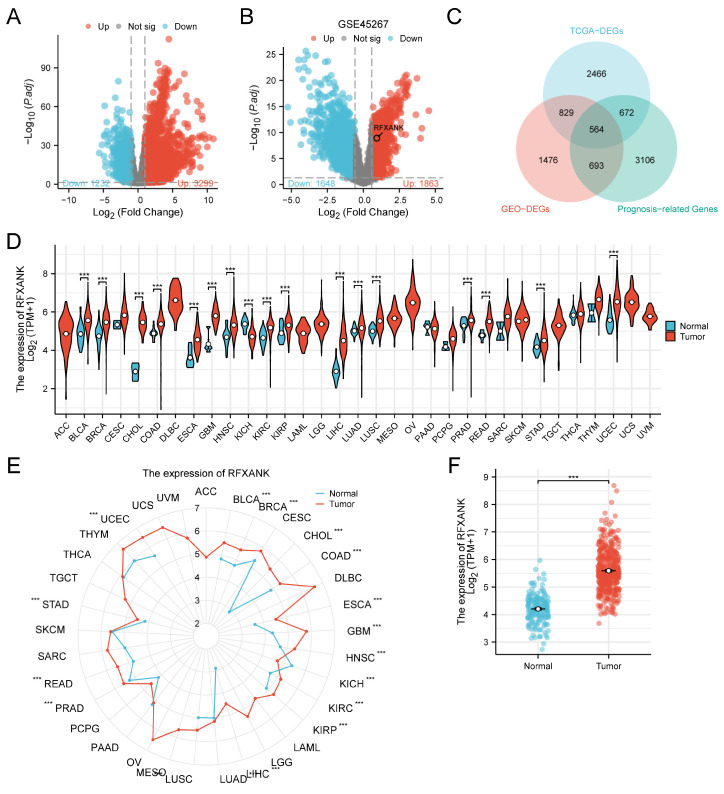
Screening and Identification of Target Genes. (**A**) Volcano plot of differentially expressed mRNAs from TCGA. (**B**) Volcano plot of differentially expressed mRNAs from GEO. (**C**) Venn diagram of TCGA-DEGs, GEO-DEGs, and prognosis-associated genes. (**D**,**E**) Differential expression of *RFXANK* in 33 tumours from the TCGA database. (**F**) Differential expression of *RFXANK* in unpaired LIHC samples. Significance indicators: ***, *p* < 0.001.

In an effort to pinpoint pivotal candidate oncogenes driving the pathogenesis and malignant evolution of HCC, we executed a comparative transcriptomic analysis utilizing datasets retrieved from the TCGA-LIHC and GEO (GSE45267) databases. As shown in the volcano plots (Figure 1A,B), the results indicated the presence of 4531 differentially expressed genes (DEGs) in TCGA-LIHC (Figure 1A) and 3511 DEGs in the GEO HCC dataset (GSE45267) (Figure 1B). Subsequently, an intersection analysis was conducted among the TCGA-derived DEGs, GEO-derived DEGs, and TCGA-derived prognosis-associated genes. The Venn diagram (Figure 1C) revealed 564 common genes across the three datasets. Through comprehensive analysis and comparison, *RFXANK* was identified as the target gene. Figure 1D,E illustrate the differential expression distribution of *RFXANK* across various malignancies: Pan-cancer profiling demonstrated a pervasive upregulation of *RFXANK* across multiple malignancies, most notably in CHOL, COAD, ESCA, GBM, HNSC, LIHC, LUAD, and STAD, relative to their non-malignant counterparts. In unpaired LIHC samples, *RFXANK* expression was significantly higher than in normal samples (Figure 1F). This finding was further corroborated by the differential analysis results from GSE45267 (Figure 1B).

### 3.2. Correlation Between RFXANK Expression and Clinicopathological Parameters

**Figure 2:**
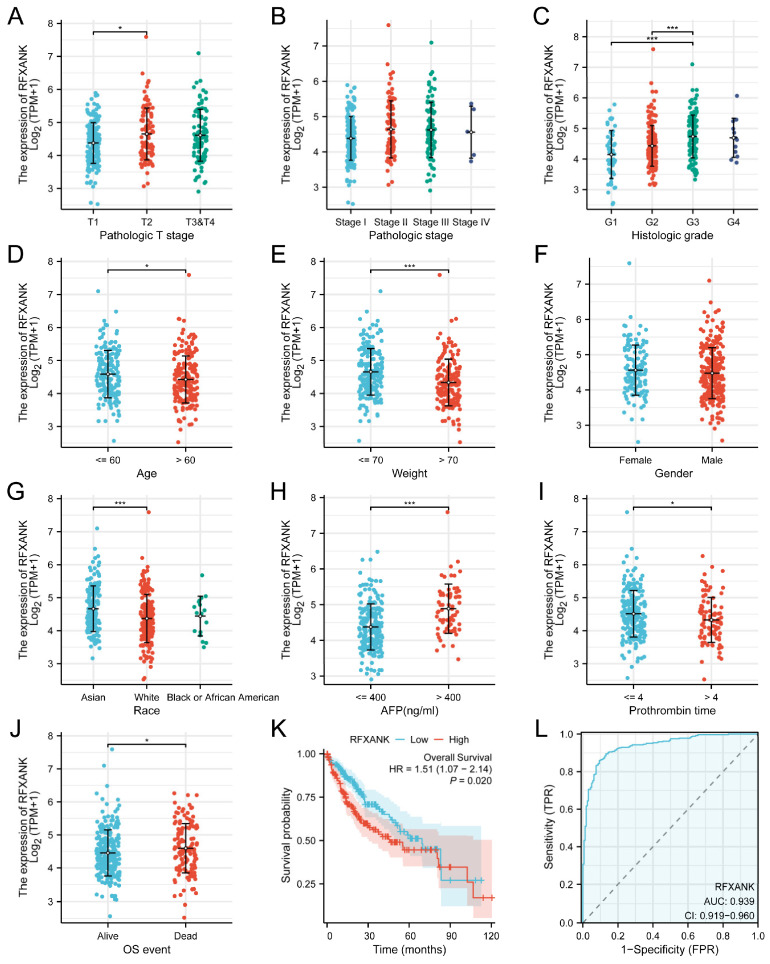
Correlation between *RFXANK* Expression and Clinicopathological Parameters. (**A**–**J**) Expression of *RFXANK* in different patient groups with different clinicopathological factors. (**K**) Overall survival curve of *RFXANK* from TCGA database. (**L**) The ROC curve of RFXANK. Significance identifier: *, *p* < 0.05; ***, *p* < 0.001.

As shown in Table 1, univariate and multivariate Cox regression analyses were performed on 14 factors, including pathological features, demographic characteristics, laboratory indices, and *RFXANK* expression levels, from 373 patients with HCC to explore their correlation with patient survival prognosis. Univariate analysis results indicated that pathological T stage (T3&T4 vs. T1: HR = 2.949, 95% CI: 1.982–4.386, *p* < 0.001), pathological M stage (M1 vs. M0: HR = 4.077, 95% CI: 1.281–12.973, *p* = 0.017), and *RFXANK* expression level (high vs. low: HR = 1.508, 95% CI: 1.066–2.135, *p* = 0.020) were significantly associated with patient survival. In contrast, pathological N stage, gender, race, age, weight, histological type, residual tumor status, histological grade, alpha-fetoprotein (AFP) level, albumin level, prothrombin time, and degree of inflammation in adjacent liver tissue showed no significant association with survival prognosis (all *p* > 0.05). Factors with *p* < 0.1 in the univariate analysis were included in a multivariate Cox regression model to adjust for confounding variables. The results revealed that pathological T stage (T3&T4 vs. T1: HR = 3.224, 95% CI: 1.956–5.316, *p* < 0.001) and *RFXANK* expression level (high vs. low: HR = 1.871, 95% CI: 1.197–2.925, *p* = 0.006) were independent risk factors affecting the survival prognosis of HCC patients. Furthermore, the correlation between *RFXANK* expression and clinicopathological factors was analyzed using the Kruskal–Wallis test and Dunn’s test. As shown in Figure 2A–J, *RFXANK* expression was significantly correlated with pathological T stage, histological grade, age, weight, gender, race, AFP level, prothrombin time, and overall survival (OS) events (*p* < 0.05). To evaluate the clinical value of *RFXANK* in LIHC, the relationship between *RFXANK* expression and patient prognosis was investigated using the TCGA-LIHC cohort. Kaplan–Meier survival curve analysis (Figure 2K) demonstrated that high expression of *RFXANK* was significantly associated with poor prognosis in patients with HCC; the overall survival in the high-expression group was significantly shorter than that in the low-expression group (HR = 1.51, 95% CI: 1.07–2.14, *p* = 0.02). Receiver operating characteristic (ROC) curve analysis (Figure 2L) revealed an area under the curve (AUC) of 0.939, suggesting that *RFXANK* expression has strong diagnostic value in patients with HCC.

### 3.3. Subgroup Analysis of RFXANK Expression and Survival Prognosis

**Figure 3:**
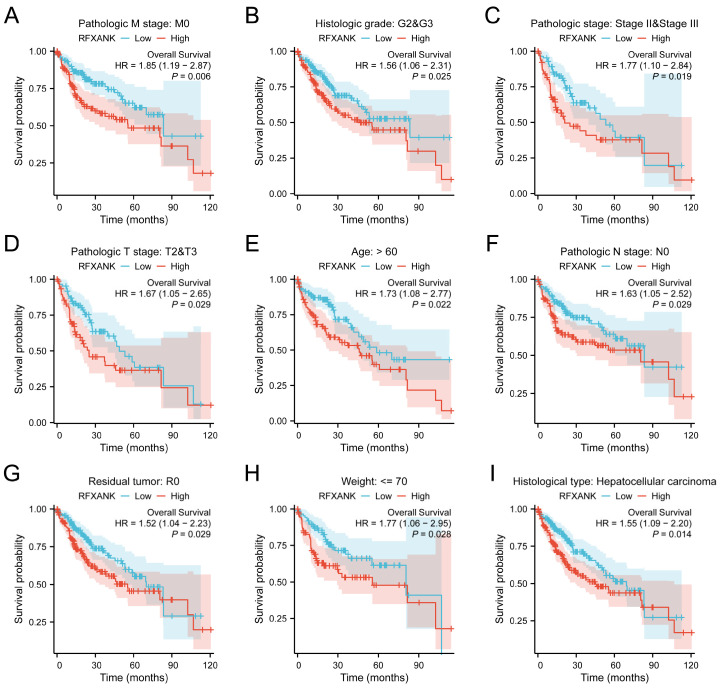
Subgroup Analysis of *RFXANK* Expression and Survival Prognosis. (**A**–**I**) Kaplan–Meier survival curves of *RFXANK* expression in relation to overall survival (OS) in different patient subgroups with different clinicopathological factors.

Figure 3A–I illustrate the survival outcomes of patients with high or low *RFXANK* expression across different HCC subgroups. In the following subgroups, patients with high *RFXANK* expression exhibited a significantly lower overall survival rate compared to those with low *RFXANK* expression, with all differences being statistically significant (*p* < 0.05): pathological M0 stage (HR = 1.85, 95% CI: 1.19–2.87, *p* = 0.006), histological grade G2 and G3 (HR = 1.56, 95% CI: 1.06–2.31, *p* = 0.025), pathological stage II and III (HR = 1.77, 95% CI: 1.10–2.84, *p* = 0.019), pathological T2 and T3 stage (HR = 1.67, 95% CI: 1.05–2.65, *p* = 0.029), age > 60 years (HR = 1.73, 95% CI: 1.08–2.77, *p* = 0.022), pathological N0 stage (HR = 1.63, 95% CI: 1.05–2.52, *p* = 0.029), R0 resection status (HR = 1.52, 95% CI: 1.04–2.23, *p* = 0.029), body weight ≤ 70 kg (HR = 1.77, 95% CI: 1.06–2.95, *p* = 0.028), and the HCC histological type (HR = 1.55, 95% CI: 1.09–2.20, *p* = 0.014). These results suggest that *RFXANK* holds promise as a novel biomarker for diagnostic evaluation in patients with HCC, providing a basis for clinical diagnostic assessment and the formulation of individualized treatment strategies.

### 3.4. Enrichment Analysis

**Figure 4:**
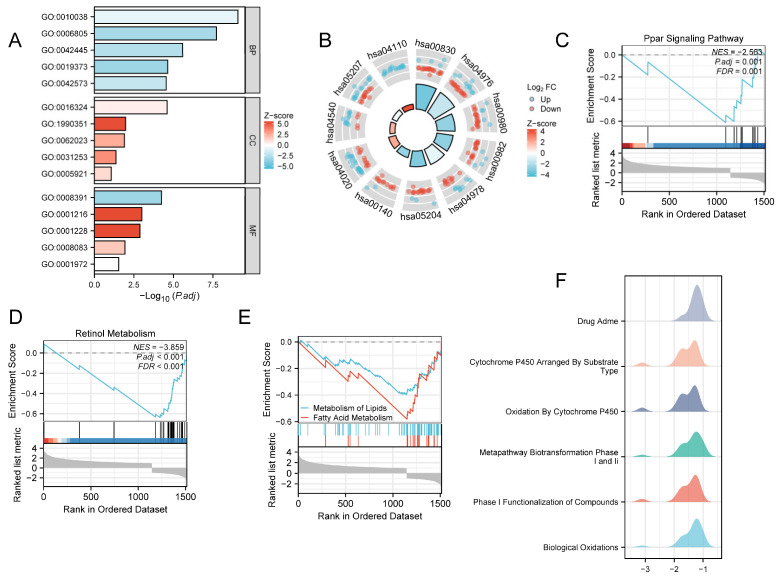
Enrichment Analysis. (**A**) Results of GO analysis. (**B**) Results of KEGG analysis. (**C**–**F**) Results of GSEA analysis.

We performed GO, KEGG, and GSEA based on single-gene differential expression analysis results, with all outcomes presented in Figure 4. GO enrichment analysis results for LIHC, detailed in Figure 4A and Table 2, revealed significant enrichment in three functional categories. At the biological process (BP) level, DEGs were notably enriched in response to metal ions, xenobiotic metabolic processes, hormone metabolic processes, epoxygenase P450 pathway, and retinoic acid metabolic processes. For the cellular component (CC) category, significant enrichment was observed in the apical plasma membrane, transporter complex, collagen-containing extracellular matrix, cell projection membrane, and gap junction. Regarding molecular function (MF), the DEGs were significantly enriched in arachidonic acid monooxygenase activity, DNA-binding transcription activator activity, RNA polymerase II-specific DNA-binding transcription activator activity, growth factor activity, and retinoic acid binding. KEGG pathway enrichment analysis results, shown in Figure 4B and Table 3, indicated that *RFXANK* is closely associated with multiple signaling pathways, including Retinol metabolism, Bile secretion, Metabolism of xenobiotics by cytochrome P450, Drug metabolism—cytochrome P450, Mineral absorption, Chemical carcinogenesis—DNA adducts, Steroid hormone biosynthesis, Calcium signaling pathway, Gap junction, Chemical carcinogenesis—receptor activation, and Cell cycle. The Z-score was used to quantify the correlation between *RFXANK* and these pathways: a negative Z-score indicates a negative correlation, while a positive Z-score denotes a positive correlation. GSEA further confirmed that DEGs were significantly enriched in signaling pathways closely linked to LIHC initiation and progression, such as the PPAR signalling pathway (Figure 4C), Retinol metabolism (Figure 4D), Metabolism of lipid, and Fatty acid metabolism (Figure 4E). Additionally, GSEA results demonstrated significant enrichment of relevant genes in biological oxidations, phase I functionalisation of compounds, metapathway biotransformation phase I and II, oxidation by cytochrome P450, cytochrome P450 arranged by substrate type, and drug ADME pathways (Figure 4F).

### 3.5. Correlation Analysis

**Figure 5:**
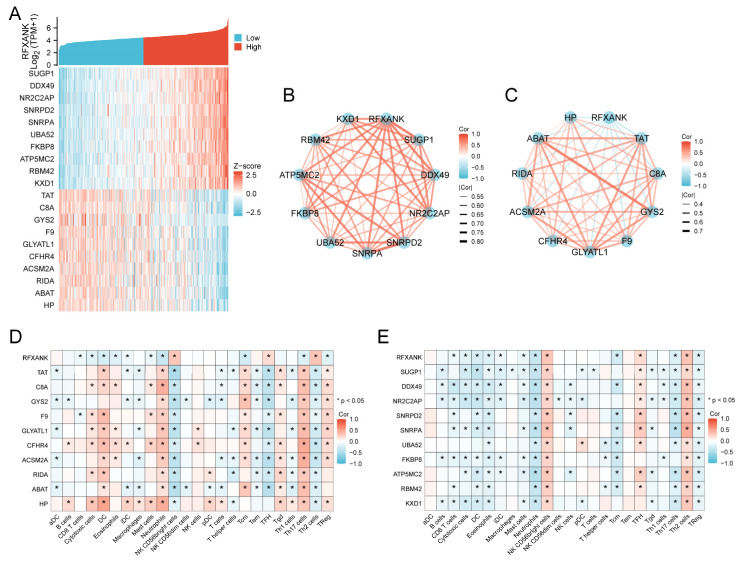
Correlation Analysis. (**A**) Co-expression heatmap of the top 10 genes positively and negatively correlated with *RFXANK*. (**B**,**C**) Correlation network diagrams of *RFXANK* with its top 10 positively and negatively correlated genes. (**D**,**E**) Heatmaps of immune cell infiltration for *RFXANK* and its top 10 positively and negatively correlated genes. Significance identifier: *, *p* < 0.05.

A single-gene correlation analysis was performed using *RFXANK* as the primary variable on the TCGA-LIHC dataset. As shown in Figure 5A, a co-expression heatmap is presented for *RFXANK* and its top 10 positively correlated genes (SUGP1, DDX49, NR2C2AP, SNRPD2, SNRPA, UBA52, FKBP8, ATP5MC2, RBM42, KXD1) and top 10 negatively correlated genes (TAT, C8A, GYS2, F9, GLYATL1, CFHR4, ACSM2A, RIDA, ABAT, HP). Correlation network diagrams (Figure 5B,C) illustrate the pairwise correlations among the top 10 positively and negatively correlated genes, respectively, including *RFXANK*. Immune infiltration association analysis further revealed that the negatively correlated core gene set exhibited a general positive correlation trend with the infiltration levels of various immune cells (Figure 5D). Among these, HP demonstrated the most significant immune-positive correlation characteristics, showing significant positive correlations (*p* < 0.05) with the infiltration levels of dendritic cells (DCs), neutrophils, macrophages, and various T-cell subsets (e.g., Tcm, Tem, TReg). In contrast, the positively correlated core gene set displayed a distinct “immune polarization” profile (Figure 5E). Genes within this set (e.g., SUGP1, DDX49, UBA52) showed significant positive correlations with the infiltration levels of NK CD56bright cells, TFH cells, and Th2 cells, but were generally significantly negatively correlated with the infiltration levels of CD8 T cells, cytotoxic cells, DCs, and Th17 cells.

### 3.6. Immune Infiltration Analysis and Correlation with Immune Checkpoints

**Figure 6:**
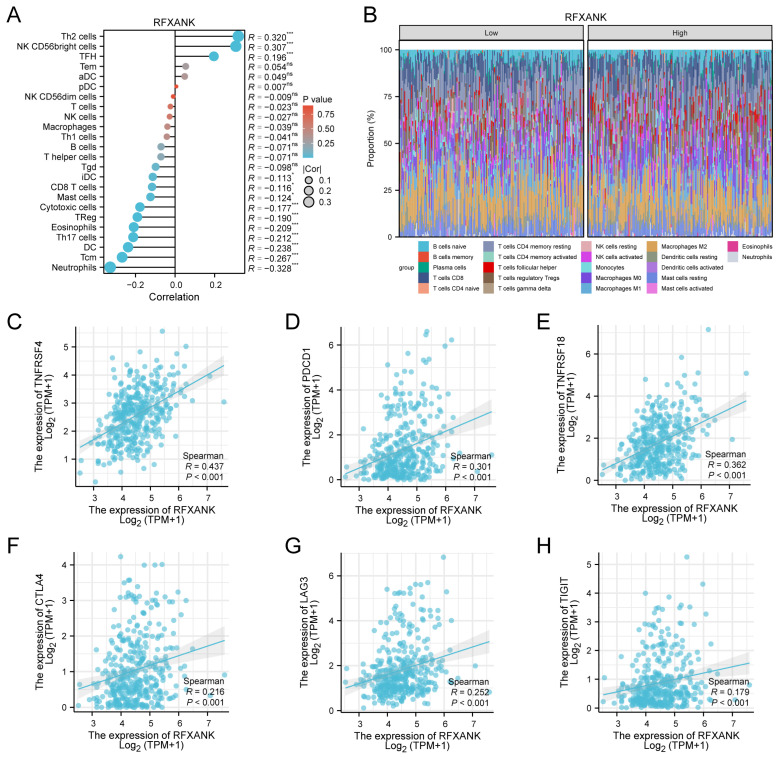
Immune Infiltration Analysis and Correlation with Immune Checkpoints. (**A**) Correlation between *RFXANK* expression and the infiltration levels of 24 immune cell types. (**B**) Proportional composition of various immune cell types in the low-expression and high-expression groups of *RFXANK*. (**C**) *RFXANK* was significantly associated with TNFRSF4. (**D**) *RFXANK* was significantly associated with PDCD1. (**E**) *RFXANK* was significantly associated with TNFRSF18. (**F**) *RFXANK* was significantly associated with CTLA4. (**G**) *RFXANK* was significantly associated with LAG-3. (**H**) *RFXANK* was significantly associated with TIGIT. Significance identifier: *, *p* < 0.05; ***, *p* < 0.001.

An analysis of the correlation between *RFXANK* expression and immune characteristics in LIHC revealed the following findings regarding immune cell infiltration (Figure 6A and Supplementary Table S1). The expression level of *RFXANK* showed significant positive correlations with Th2 cells (R = 0.320, q < 0.001), NK CD56bright cells (R = 0.307, q < 0.001), and TFH cells (R = 0.196, q < 0.001). Conversely, significant negative correlations were observed with Neutrophils (R = −0.328, q < 0.001), Tcm cells (R = −0.267, q < 0.001), DC (R = −0.238, q < 0.001), Th17 cells (R = −0.212, q < 0.001), Eosinophils (R = −0.209, q < 0.001), TReg cells (R = −0.190, q < 0.001), Cytotoxic cells (R = −0.177, *p* = 0.001), Mast cells (R = −0.124, *p* = 0.035), and CD8 T cells (R = −0.116, *p* = 0.049). Figure 6B further illustrates the differential distribution patterns of 22 immune cell types between the *RFXANK* high-expression and low-expression groups. The results of the immune checkpoint correlation analysis (Figure 6C–H) demonstrated that *RFXANK* expression was significantly positively correlated with several key immune checkpoint molecules, including TNFRSF4 (R = 0.437), TNFRSF18 (R = 0.362), PDCD1 (PD-1, R = 0.301), LAG3 (R = 0.252), CTLA4 (R = 0.216), and TIGIT (R = 0.179) (*p* < 0.001).

### 3.7. Effects of RFXANK Knockdown on Hepatocellular Carcinoma Cells

**Figure 7:**
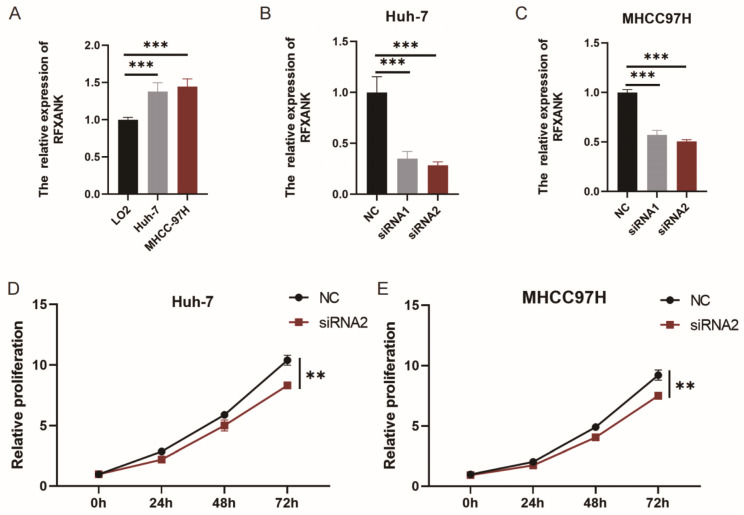

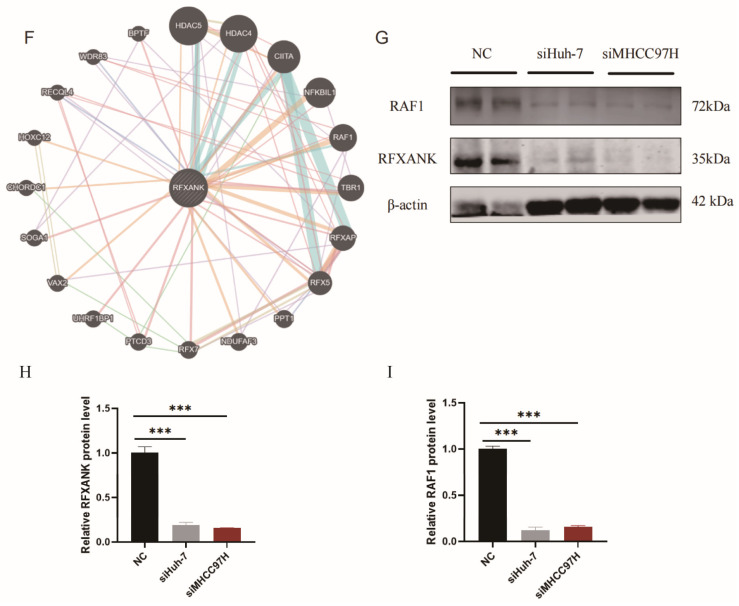
Effects of *RFXANK* Knockdown on Hepatocellular Carcinoma Cells. (**A**) Relative mRNA expression levels of *RFXANK* in normal hepatocyte line LO2 and hepatocellular carcinoma cell lines Huh-7 and MHCC-97H. (**B**) Knockdown efficiency of *RFXANK* in Huh-7 cells. (**C**) Knockdown efficiency of *RFXANK* in MHCC97H cells. (**D**,**E**) Proliferation of Huh-7 and MHCC97H cells assessed by CCK-8 assay. (**F**) RFXANK protein interaction network diagram. (**G**–**I**) Western blot analysis of RAF1 and RFXANK protein levels following *RFXANK* knockdown in Huh-7 and MHCC97H cells. Significance identifier: **, *p* < 0.01, ***, *p* < 0.001. All results were from three independent experiments (*n* = 3).

The relative mRNA expression levels of *RFXANK* were measured in the normal hepatocyte cell line LO2 and the HCC cell lines (Huh-7 and MHCC97H). As shown in Figure 7A, the mRNA expression of *RFXANK* was significantly higher in Huh-7 and MHCC97H cells compared to LO2 cells. To elucidate the role of the *RFXANK* gene in HCC cell proliferation, *RFXANK* expression was silenced using siRNAs (siRNA1, siRNA2) in both Huh-7 and MHCC97H cell lines, with a non-targeting siRNA serving as the negative control (NC). Cell proliferation activity was assessed at different time points (0 h, 24 h, 48 h, 72 h). As shown in Figure 7B,C, both siRNA1 and siRNA2 significantly reduced *RFXANK* expression compared to the negative control, with siRNA2 demonstrating greater interference efficiency than siRNA1. Therefore, siRNA2 was selected for subsequent experiments due to its higher efficiency. The results in Figure 7D,E showed that the proliferation of both Huh-7 and MHCC97H cells transfected with *RFXANK* siRNA2 was significantly reduced compared with the negative control group (*p* < 0.05). As indicated in Figure 7F, RFXANK may potentially interact with genes such as HDAC5, HDAC4, CIITA, NFKBIL1, RAF1, TBR1, RFXAP, RFX5, PPT1, NDUFAF3, RFX7, PTCD3, UHRFBP1, VAX2, SOGA1, CHORDC1, HOXC12, RECQL4, WDR83, and BPIF. The results in Figure 7G demonstrated that, compared to the negative control group, the protein expression levels of RFXANK were significantly downregulated in Huh-7 (siHuh-7) and MHCC97H (siMHCC97H) cells transfected with *RFXANK* siRNA2. Concurrently, the expression level of RAF1 protein was also markedly reduced (Figure 7G–I).

## 4. Discussion

HCC emerges and advances through intricate, multi-step biological trajectories driven by a constellation of oncogenic factors. Notwithstanding notable breakthroughs in diagnostic modalities and therapeutic interventions, the high heterogeneity, propensity for recurrence, and complex tumor microenvironment of HCC mean that existing clinical staging systems and single biomarkers remain insufficient for predicting individualized prognosis, lacking adequate sensitivity and specificity. Therefore, identifying key molecules capable of simultaneously untangling the malignant biological behavior of tumors and the characteristics of the immune microenvironment has become crucial for overcoming the bottlenecks in HCC diagnosis and treatment. Through the integration of multi-cohort transcriptomic profiles alongside exhaustive in vitro assays, our research provides the first systematic evidence establishing *RFXANK* as a robust independent indicator of HCC diagnosis. By integrating multi-centre transcriptomic data with in vitro biological experiments, this study has, for the first time, systematically demonstrated the clinical value of *RFXANK* as an independent diagnostic marker for HCC. It has also preliminarily elucidated the correlation between changes in *RFXANK* expression and the cell cycle, tumour cell proliferation, and the characteristics of the immunosuppressive microenvironment, thereby providing a novel perspective on the mechanisms underlying HCC progression and identifying new therapeutic targets.

In previous studies, *RFXANK* has been primarily defined as a specific transcriptional regulator of the promoter region of MHC class II molecule genes [16], with its dysfunction often associated with primary immunodeficiency diseases [18]. However, in recent years, aberrant expression of RFX family members in tumors has gradually garnered attention [19,20,21,22,23]. Cross-database mining of the TCGA and GEO databases unveiled a pervasive upregulation of *RFXANK* across a broad spectrum of malignancies, with a particularly marked elevation observed in HCC tissues. Its high expression is closely correlated with patients’ pathological T stage, histological grade, and AFP levels. Multivariate Cox proportional hazards modeling ascertained that high *RFXANK* expression serves as an independent detrimental factor for the OS of individuals with HCC. Results from ROC curve analysis further corroborated *RFXANK*’s superior predictive performance as a biomarker. Furthermore, subgroup analysis demonstrated that *RFXANK* maintains consistent diagnostic value across different clinicopathological characteristics, suggesting that *RFXANK* may be a diagnostic biomarker with high clinical translation potential.

Our in vitro assays revealed that the silencing of *RFXANK* substantially impaired the proliferative capacity of Huh-7 and MHCC97H cell lines, providing compelling evidence for its oncogenic role in HCC. Additionally, our study revealed a marked decrease in RAF1 protein expression following *RFXANK* silencing. As a member of the RAF kinase family, RAF1 serves as a key component of the MAPK/ERK signaling pathway. It plays a central role in tumor cell growth, transformation, and anti-apoptosis [39,40,41]. Given that *RFXANK* contains highly conserved ankyrin repeat domains and protein interaction network predictions suggest a potential association with RAF1, coupled with the observation in this study that “knockdown of *RFXANK* significantly downregulates RAF1 protein levels,” we hypothesize that the role of *RFXANK* in promoting malignant proliferation in HCC may be related to the expression levels of RAF1. Multi-dimensional analyses, including GO, KEGG, co-expression network, and GSEA, delineated the regulatory role of *RFXANK* in HCC initiation and progression, highlighting its potential as a core regulator of HCC metabolic reprogramming and phenotypic dedifferentiation. Consistent results from GO and KEGG enrichment analyses showed that *RFXANK*-associated DEGs were significantly enriched in pathways such as the cytochrome P450 pathway, xenobiotic metabolism, and retinol metabolism. The CYP450 enzyme system, a key player in xenobiotic and drug metabolism, can contribute to tumorigenesis through mechanisms such as procarcinogen activation when dysregulated [42,43]. Furthermore, GSEA indicated that these DEGs prominently modulate the mitotic cycle alongside essential metabolic routes, most notably the PPAR signaling axis and lipid/retinol biotransformation. Accumulating evidence indicates that dysregulation of both the PPAR signaling pathway and fatty acid metabolism is closely linked to tumor development: aberrant activation of the PPAR pathway modulates tumor cell proliferation, invasion, and metastasis [44,45], while fatty acid metabolism disorders reshape lipid homeostasis to meet the demands of malignant tumor proliferation and tumor microenvironment remodeling [46]. Single-gene correlation analysis further confirmed that *RFXANK* drives the “dedifferentiation” malignant phenotype of HCC cells. Key genes maintaining normal hepatocellular physiological functions, such as TAT and GYS2 [47,48], were significantly negatively correlated with *RFXANK* expression, suggesting the loss of mature hepatocellular characteristics in HCC cells. Notably, previous studies have validated that low GYS2 expression is associated with poor prognosis in HCC [48]. In contrast, genes positively correlated with *RFXANK* expression—including SNRPA, SNRPD2, and DDX49—are all closely implicated in HCC progression. Specifically, SNRPA promotes HCC tumor growth and enhances drug resistance [49]; elevated SNRPD2 levels are prevalent in hepatic malignancies and are closely tied to advanced disease stages and suboptimal patient survival [50]; DDX49 is upregulated in HCC tissues, and its knockdown markedly suppresses HCC cell formation, metastasis, and tumor growth [51]. Based on the above findings, we reasonably infer that abnormal expression of *RFXANK* may be associated with alterations in the cell cycle and the lipid metabolic network of tumour cells, with multiple pathways acting in concert to promote the development of HCC.

Data from the immune infiltration assessment indicated a robust positive association between elevated *RFXANK* levels and the presence of Th2 cells, TFH, and low-cytotoxicity CD56bright NK cells, while showing significant negative correlations with neutrophils, Tcm, DC, Th17 cells, TReg, cytotoxic cells, mast cells, CD8 T cells, and iDC. Immune cell infiltration is associated with improved patient prognosis, whereas low infiltration levels may facilitate immune evasion by cancer cells, leading to poor outcomes [52]. During tumor progression, reduced recruitment of neutrophils and DCs indicates impaired antigen presentation and weakened anti-tumor inflammatory responses, rendering the tumor microenvironment less capable of effectively recognizing cancer cells and thereby promoting tumor cell migration, invasion, and growth [53]. The immune infiltration pattern characterized by “Th2 cell accumulation and Th1 deficiency” suggests that *RFXANK* drives the TME to shift from an anti-tumor cellular immune state toward a pro-tumor immune-tolerant state [54]. When Th2 cell infiltration is elevated, cytokines such as IL-4 and IL-10 are secreted, antagonizing Th1-type immune responses and suppressing the cytotoxic function of CD8^+^ T cells. Consequently, recalibrating the Th1/Th2 ratio represents a pivotal strategy for enhancing the efficacy of oncological interventions [54]. Furthermore, the immunoregulatory balance between lymphocyte subsets and non-lymphocytic cells may also be disrupted; such interference readily induces an immunosuppressive state, accelerates malignant tumor progression, and undermines the efficacy of immunotherapy [55]. iDCs can promote T-cell activation, and NK cells exert a pivotal regulatory influence on the immune response via their crosstalk with DCs [56]. However, decreased infiltration of iDCs and DCs leads to weakened anti-tumor immunity. Meanwhile, although CD56bright NK cell infiltration increases, its weak cytotoxicity and functional limitations may allow tumor cells to evade effective immune surveillance [57]. In summary, we reasonably infer that upregulation of *RFXANK* may significantly suppress anti-tumour immune responses in patients by remodelling the immune microenvironment, thereby promoting tumour progression.

We also revealed that *RFXANK* expression was significantly positively correlated with classical immune checkpoint molecules, including PD-1, CTLA4, LAG3, and TIGIT, and was closely associated with TNFRSF4 and TNFRSF18. Dysregulated TNFRSF4 signaling can lead to T-cell dysfunction and impair immune surveillance [58]. By stimulating effector T cell populations while simultaneously blunting Treg suppressive capacity, TNFRSF18 bolsters anti-tumor defense mechanisms. The PD-1/PD-L1 axis, LAG3, CTLA4, and TIGIT collectively constitute a critical immunosuppressive barrier [59]. Among these, PD-1 and CTLA4 primarily inhibit T-cell activation and effector functions directly [60,61]. LAG3 mediates immunosuppression by blocking co-stimulatory signaling pathways [62]. TIGIT suppresses effector cell functions while simultaneously enhancing Treg activity, jointly promoting tumor immune evasion [63]. These findings identify *RFXANK* as a potential predictive biomarker for response to immune checkpoint blockade therapy.

In summary, silencing *RFXANK* reduced RAF1 protein levels and *RFXANK* expression correlated with cell proliferation, metabolic reprogramming, and features of the tumor immune microenvironment. These observations collectively suggest that *RFXANK* is linked to multiple biological processes associated with HCC development. *RFXANK* emerges as a compelling candidate for both innovative diagnostic screening and targeted intervention in HCC management.

### Limitations

However, this study has certain limitations: the data were derived from public databases and tissue-level transcriptomic analyses, and thus we could not rule out potential confounding effects of HBV/HCV infection status; furthermore, the cellular origin of *RFXANK* expression and associated phenotypes could not be clearly identified, and its specific functions in tumour cells and immune cells require further validation through single-cell sequencing and cellular functional assays. Furthermore, whilst this study demonstrates that *RFXANK* influences RAF1 protein expression through in vitro loss-of-function experiments, the mechanism of interaction between the two has not yet been directly validated. Key questions regarding the specific binding mode and binding site of *RFXANK* and RAF1, as well as whether RAF1 protein levels are regulated by inhibiting ubiquitin-mediated degradation, require elucidation through molecular interaction experiments, including co-immunoprecipitation, GST precipitation, ubiquitination assays, and protein stability analyses. In summary, the conclusions of this study require further validation through additional in vitro and in vivo experiments to clarify the molecular mechanisms by which *RFXANK* promotes the development and progression of HCC.

## 5. Conclusions

To sum up, *RFXANK* was significantly overexpressed in HCC tissues and was closely associated with adverse clinical indicators, including pathological T stage and histological grade. *RFXANK* was identified as an independent risk factor for patient survival prognosis. In vitro experiments further confirmed that knocking down *RFXANK* significantly inhibited the proliferative capacity of Huh-7 and MHCC97H cells and downregulated RAF1 expression. Furthermore, *RFXANK* expression showed a significant positive correlation with immune checkpoint molecules such as PD-1 and CTLA4, as well as with the infiltration of various inhibitory immune cells. This suggests that *RFXANK* may promote tumor progression by regulating metabolic reprogramming and fostering an immunosuppressive tumor microenvironment. In summary, *RFXANK* drives HCC development through a multi-dimensional mechanism involving “proliferation drive, metabolic remodeling, and immune evasion,” positioning it as a promising novel biomarker and potential therapeutic target for the precise diagnosis and treatment of HCC.

## Figures and Tables

**Table 1:** Univariate and multivariate analyses of clinicopathological parameters in patients with hepatocellular carcinoma.

Characteristics	Total (N)	Univariate Analysis		Multivariate Analysis	
		Hazard Ratio (95% CI)	*p* Value	Hazard Ratio (95% CI)	*p* Value
Pathologic T stage	370				
T1	183	Reference		Reference	
T2	94	1.428 (0.901–2.264)	0.129	1.543 (0.857–2.778)	0.149
T3&T4	93	2.949 (1.982–4.386)	<0.001	3.224 (1.956–5.316)	<0.001
Pathologic N stage	258				
N0	254	Reference			
N1	4	2.029 (0.497–8.281)	0.324		
Pathologic M stage	272				
M0	268	Reference		Reference	
M1	4	4.077 (1.281–12.973)	0.017	1.889 (0.578–6.171)	0.292
Gender	373				
Female	121	Reference			
Male	252	0.793 (0.557–1.130)	0.200		
Race	344				
Asian	159	Reference			
White	185	1.324 (0.909–1.928)	0.144		
Age	373				
≤60	177	Reference			
>60	196	1.205 (0.850–1.708)	0.295		
Weight	345				
≤70	184	Reference			
>70	161	0.941 (0.657–1.346)	0.738		
Histological type	373				
Hepatocellular carcinoma	363	Reference			
Hepatocholangio carcinoma (mixed) Fibrolamellar carcinoma	10	0.439 (0.061–3.145)	0.412		
Residual tumor	344				
R0	326	Reference			
R1&R2	18	1.604 (0.812–3.169)	0.174		
Histologic grade	368				
G1	55	Reference			
G2	178	1.162 (0.686–1.969)	0.576		
G3	123	1.185 (0.683–2.057)	0.545		
G4	12	1.681 (0.621–4.549)	0.307		
AFP (ng/mL)	279				
≤400	215	Reference			
>400	64	1.075 (0.658–1.759)	0.772		
Albumin (g/dL)	299				
<3.5	69	Reference			
≥3.5	230	0.897 (0.549–1.464)	0.662		
Prothrombin time	296				
≤4	207	Reference			
>4	89	1.335 (0.881–2.023)	0.174		
Adjacent hepatic tissue inflammation	236				
None	118	Reference			
Mild&Severe	118	1.194 (0.734–1.942)	0.475		
*RFXANK*	373				
Low	187	Reference		Reference	
High	186	1.508 (1.066–2.135)	0.020	1.871 (1.197–2.925)	0.006

**Table 2:** GO analysis.

Ontology	ID	Description	GeneRatio	BgRatio	*p* Value	p.adjust	Z-Score
BP	GO:0010038	response to metal ion	65/1324	351/18,800	4.93 × 10^−13^	8.42 × 10^−10^	−0.8682431
BP	GO:0006805	xenobiotic metabolic process	30/1324	108/18,800	4.04 × 10^−11^	1.88 × 10^−8^	−2.9211870
BP	GO:0042445	hormone metabolic process	42/1324	230/18,800	1.02 × 10^−8^	2.61 × 10^−6^	−2.4688536
BP	GO:0019373	epoxygenase P450 pathway	10/1324	19/18,800	1.49 × 10^−7^	2.31 × 10^−5^	−3.1622777
BP	GO:0042573	retinoic acid metabolic process	13/1324	34/18,800	2.27 × 10^−7^	2.87 × 10^−5^	−1.9414507
CC	GO:0016324	apical plasma membrane	56/1430	358/19,594	4.73 × 10^−8^	2.52 × 10^−5^	0.5345225
CC	GO:1990351	transporter complex	48/1430	399/19,594	0.0004	0.0106	5.1961524
CC	GO:0062023	collagen-containing extracellular matrix	50/1430	429/19,594	0.0007	0.0126	2.5455844
CC	GO:0031253	cell projection membrane	39/1430	339/19,594	0.0032	0.0426	3.3626912
CC	GO:0005921	gap junction	7/1430	32/19,594	0.0073	0.0857	1.1338934
MF	GO:0008391	arachidonic acid monooxygenase activity	10/1361	21/18,410	7.82 × 10^−7^	5.71 × 10^−5^	−3.1622777
MF	GO:0001216	DNA-binding transcription activator activity	59/1361	466/18,410	3.4 × 10^−5^	0.0010	5.5981232
MF	GO:0001228	DNA-binding transcription activator activity, RNA polymerase II-specific	58/1361	462/18,410	5.04 × 10^−5^	0.0013	5.5148702
MF	GO:0008083	growth factor activity	24/1361	162/18,410	0.0008	0.0117	1.6329932
MF	GO:0001972	retinoic acid binding	6/1361	20/18,410	0.0025	0.0287	0.0000000

**Table 3:** KEGG analysis.

Ontology	ID	Description	GeneRatio	BgRatio	*p* Value	p.adjust	Z-Score
KEGG	hsa00830	Retinol metabolism	25/604	68/8164	4.22 × 10^−12^	6.5 × 10^−10^	−3.4000000
KEGG	hsa04976	Bile secretion	28/604	89/8164	1.7 × 10^−11^	1.75 × 10^−9^	−1.5118579
KEGG	hsa00980	Metabolism of xenobiotics by cytochrome P450	23/604	78/8164	4.64 × 10^−9^	3.57 × 10^−7^	−2.7106874
KEGG	hsa00982	Drug metabolism—cytochrome P450	21/604	72/8164	2.71 × 10^−8^	1.46 × 10^−6^	−2.8368326
KEGG	hsa04978	Mineral absorption	19/604	60/8164	2.84 × 10^−8^	1.46 × 10^−6^	−0.6882472
KEGG	hsa05204	Chemical carcinogenesis—DNA adducts	20/604	69/8164	6.53 × 10^−8^	2.87 × 10^−6^	−3.1304952
KEGG	hsa00140	Steroid hormone biosynthesis	14/604	61/8164	0.0001	0.0038	−2.6726124
KEGG	hsa04020	Calcium signaling pathway	34/604	240/8164	0.0002	0.0052	2.0579830
KEGG	hsa04540	Gap junction	15/604	88/8164	0.0018	0.0357	1.8073922
KEGG	hsa05207	Chemical carcinogenesis—receptor activation	28/604	212/8164	0.0019	0.0357	−0.3779645
KEGG	hsa04110	Cell cycle	19/604	126/8164	0.0022	0.0395	4.3588989

## Data Availability

The HCC datasets used and/or analyzed in this study can be found in the following repositories: Gene Expression Omnibus (GEO, https://www.ncbi.nlm.nih.gov/geo/download/?acc=GSE45267, accessed on 25 November 2025), University of California Santa Cruz Xena (UCSC XENA, https://xenabrowser.net/datapages/, accessed on 25 November 2025), and The Cancer Genome Atlas (TCGA-LIHC, https://portal.gdc.cancer.gov/analysis_page?app=Downloads, accessed on 25 November 2025). The datasets generated during and/or analyzed during the current study are also available from the corresponding author upon reasonable request.

## Supplementary Materials

The following supporting information can be downloaded at: https://www.mdpi.com/article/10.3390/genes17040406/s1, Figure S1. Original data of WB original bands; Table S1: Analysis of the correlation between *RFXANK* expression and immune cells; File S1: The Relevant R code.
