# Supplementary material for: RFXANK: A Novel Immune-Related Biomarker for Hepatocellular Carcinoma
Source: Genes (Basel). 2026 Mar 31;17(4):406. doi: 10.3390/genes17040406 (PMC13115998; doi:10.3390/genes17040406)
Supplement: Supplementary file 1 [file genes-17-00406-s001.zip › genes-4214281-supplementary.pdf]

RFXANK: A Novel Immune-Related Biomarker for Hepatocellular Carcinoma

Figure S1. Original data of WB original bands.

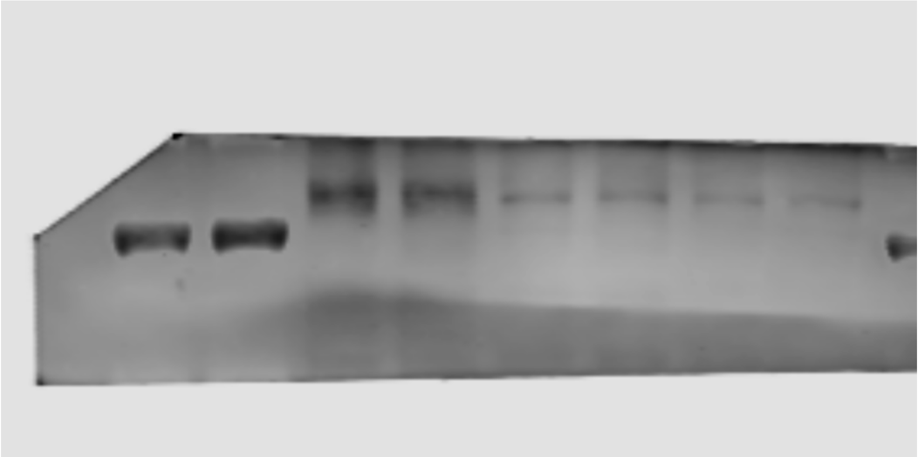

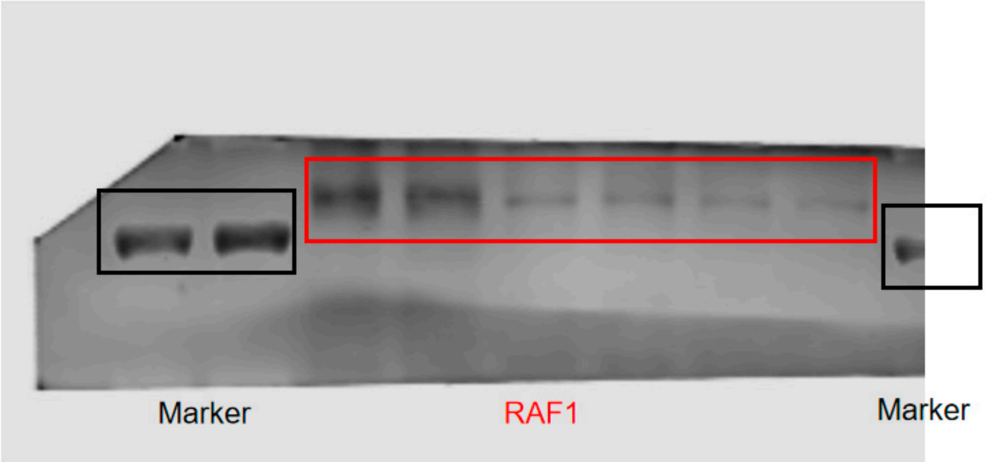

Marker: 70kDA

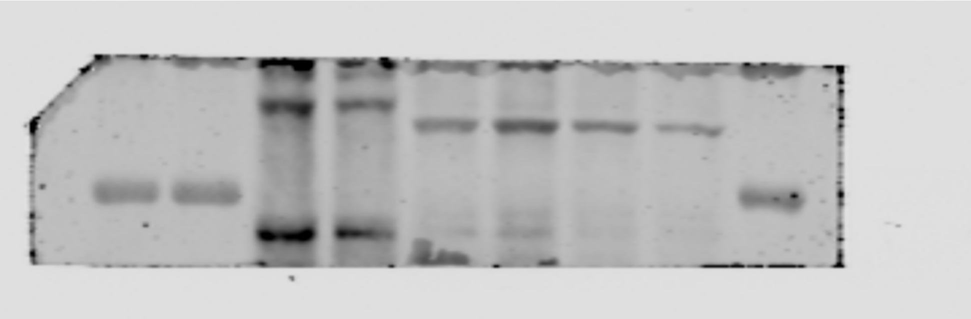

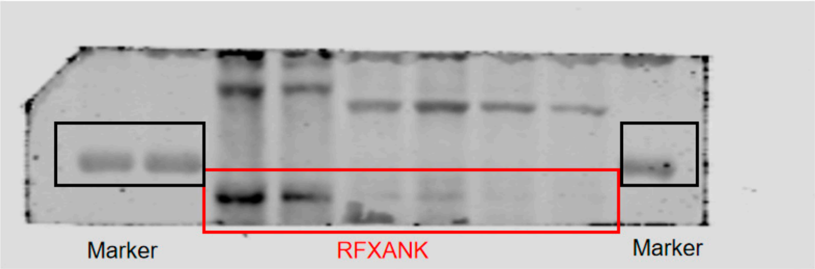

Marker: 40kDA

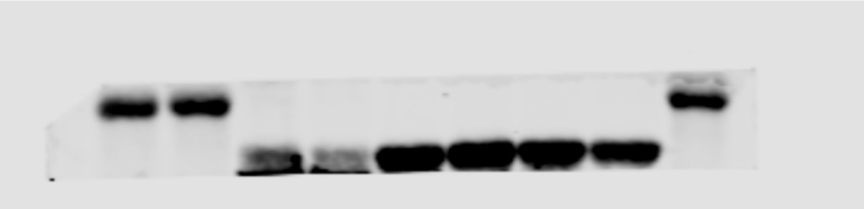

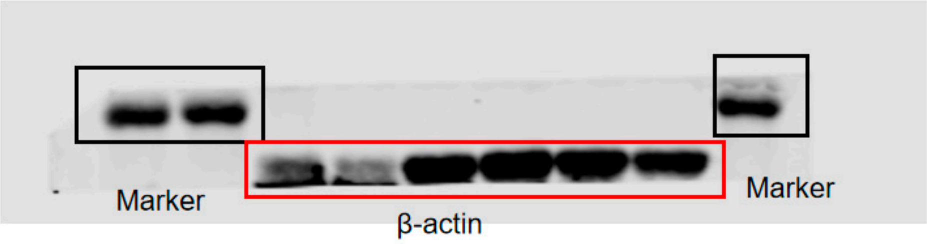

Marker: 55 kDA

Table S1: Analysis of the correlation between RFXANK expression and immune cells.

var1	var2	cor_spearman	pvalue_spearman	q_value (FDR BH)
RFXANK	aDC	0.04873	3.4735e-01	0.4413
RFXANK	B cells	-0.07077	1.7204e-01	0.2581
RFXANK	CD8 T cells	-0.11625	2.4558e-02	**0.04912**
RFXANK	Cytotoxic cells	-0.17747	5.6476e-04	**0.001355**
RFXANK	DC	-0.23752	3.4077e-06	**1.0175e-08**
RFXANK	Eosinophils	-0.20877	4.7231e-05	**0.0001483**
RFXANK	iDC	-0.11260	2.9460e-02	**0.05439**
RFXANK	Macrophages	-0.03855	4.5732e-01	0.5488
RFXANK	Mast cells	-0.12418	1.6268e-02	**0.03549**
RFXANK	Neutrophils	-0.32786	8.0453e-11	**1.9309e-09**
RFXANK	NK CD56bright cells	0.30732	1.2719e-09	**1.9309e-09**
RFXANK	NK CD56dim cells	-0.009447	8.5551e-01	0.8929
RFXANK	NK cells	-0.02734	5.9810e-01	0.6835
RFXANK	pDC	0.006648	8.9804e-01	0.8980
RFXANK	T cells	-0.02346	6.5110e-01	0.7104
RFXANK	T helper cells	-0.07141	1.6816e-01	0.2581
RFXANK	Tcm	-0.26745	1.5133e-07	**9.0800e-07**
RFXANK	Tem	0.05380	2.9944e-01	0.4228
RFXANK	TFH	0.19616	1.3448e-04	**0.0004034**
RFXANK	Tgd	-0.09813	5.7955e-02	0.09934
RFXANK	Th1 cells	-0.04149	4.2369e-01	0.5352
RFXANK	Th17 cells	-0.21158	3.7085e-05	**0.0001483**

var1	var2	cor_spearman	pvalue_spearman	q_value (FDR BH)
RFXANK	Th2 cells	0.31966	2.4824e-10	**2.9789e-09**
RFXANK	TReg	-0.19040	2.1247e-04	**0.0005666**

File S1. The Relevant R code.

```
# Load Required R Packages
library(TCGAbiolinks)
library(SummarizedExperiment)
library(DESeq2)
library(dplyr)
library(ggplot2)
library(ggrepel)
library(survival)
library(survminer)
library(timeROC)
library(pheatmap)
library(clusterProfiler)
library(org.Hs.eg.db)
library(enrichplot)
library(GSEABase)
library(GSVA)

# Set Working Directory
setwd("~/TCGA_LIHC_analysis")

#

# 1. Data Download and Preprocessing
#

query_exp <- GDCquery(
  project = "TCGA-LIHC",
  data.category = "Transcriptome Profiling",
  data.type = "Gene Expression Quantification",
  workflow.type = "STAR - Counts"
)
GDCdownload(query_exp, method = "api", files.per.chunk = 10)
```

```
exp_data <- GDCprepare(query_exp)

# Extract Expression Matrix and Clinical Information
counts_matrix <- assay(exp_data)
clinical_data <- colData(exp_data)

# Gene ID Conversion (ENSEMBL -> SYMBOL)
library(AnnotationDbi)
library(org.Hs.eg.db)
gene_ids <- rownames(counts_matrix)
gene_symbols <- mapIds(org.Hs.eg.db, keys = gene_ids, column = "SYMBOL",
                       keytype = "ENSEMBL", multiVals = "first")
keep <- !is.na(gene_symbols) & !duplicated(gene_symbols)
counts_matrix <- counts_matrix[keep, ]
rownames(counts_matrix) <- gene_symbols[keep]

# Group Information (Tumor vs Normal)
sample_type <- ifelse(colData(exp_data)$definition %in% c("Primary solid Tumor"), "Tumor",
"Normal")
coldata <- data.frame(row.names = colnames(counts_matrix), group = factor(sample_type, levels
= c("Normal", "Tumor")))

#
```

# 2. Differential Expression Analysis（DESeq2）

```
dds <- DESeqDataSetFromMatrix(countData = counts_matrix, colData = coldata, design = ~
group)
keep <- rowSums(counts(dds) >= 10) >= 10
dds <- dds[keep, ]
dds <- DESeq(dds)

res <- results(dds, contrast = c("group", "Tumor", "Normal"), alpha = 0.05, lfcThreshold = 0.9)
deg_df <- as.data.frame(res)
deg_df$gene <- rownames(deg_df)

sig_deg <- subset(deg_df, abs(log2FoldChange) > 0.9 & padj < 0.05)
cat("Number of significant DEGs:", nrow(sig_deg), "\n")

write.csv(deg_df, "LIHC_DESeq2_results.csv", row.names = FALSE)
write.csv(sig_deg, "LIHC_significant_genes.csv", row.names = FALSE)

#
```

3. Volcano Plot

```
deg_df$sig <- ifelse(deg_df$gene == "RFXANK", "RFXANK",
                     ifelse(deg_df$padj < 0.05 & abs(deg_df$log2FoldChange) > 0.9, "Sig",
"Not Sig"))

p_volcano <- ggplot(deg_df, aes(x = log2FoldChange, y = -log10(padj), color = sig)) +
  geom_point(alpha = 0.6, size = 1.2) +
  scale_color_manual(values = c("Sig" = "red", "Not Sig" = "gray", "RFXANK" = "blue")) +
  geom_vline(xintercept = c(-0.9, 0.9), linetype = "dashed", color = "black") +
  geom_hline(yintercept = -log10(0.05), linetype = "dashed", color = "black") +
  labs(title = "Volcano plot of DEGs in TCGA-LIHC",
       x = expression(log[2]("Fold Change")),
       y = expression(-log[10]("FDR"))) +
  theme_minimal() +
  theme(legend.title = element_blank()) +
  geom_text_repel(data = subset(deg_df, gene == "RFXANK"),
                  aes(label = gene), color = "blue", size = 4)

ggsave("Volcano_RFXANK.pdf", p_volcano, width = 8, height = 6)
```

4. OS Kaplan-Meier Survival Curve for RFXANK

```
vst_mat <- assay(vst(dds))
rfxank_exp <- vst_mat["RFXANK", ]

clinical <- colData(exp_data)
surv_data <- data.frame(
  sample = colnames(vst_mat),
  RFXANK = rfxank_exp,
  OS.time = as.numeric(clinical$days_to_death),
  OS = ifelse(clinical$vital_status == "Dead", 1, 0)
)
censored <- is.na(surv_data$OS.time) | surv_data$OS.time == 0
surv_data$OS.time[censored] <- as.numeric(clinical$days_to_last_follow_up[censored])
surv_data$OS <- ifelse(is.na(surv_data$OS), 0, surv_data$OS)
surv_data <- surv_data[!is.na(surv_data$OS.time) & surv_data$OS.time > 0, ]

surv_data$group <- ifelse(surv_data$RFXANK > median(surv_data$RFXANK), "High", "Low")
surv_data$group <- factor(surv_data$group, levels = c("Low", "High"))
```

```r
fit <- survfit(Surv(OS.time, OS) ~ group, data = surv_data)

p_km <- ggsurvplot(fit, data = surv_data,
                   pval = TRUE, conf.int = TRUE,
                   risk.table = TRUE,
                   xlab = "Time (days)", ylab = "Overall Survival",
                   title = "RFXANK Expression and Overall Survival in LIHC",
                   legend.title = "RFXANK",
                   palette = c("#2E9FDF", "#E7B800"))
ggsave("KM_curve_RFXANK.pdf", p_km$plot, width = 6, height = 5)

#


# 5. ROC Curve for RFXANK
#

roc_obj <- roc(surv_data$OS, surv_data$RFXANK, levels = c(0, 1), direction = "<")
auc_val <- auc(roc_obj)

# Plot ROC Curve
pdf("ROC_RFXANK.pdf", width = 5, height = 5)
plot(roc_obj, col = "blue", lwd = 2, legacy.axes = TRUE,
     main = paste0("RFXANK ROC Curve (Overall Survival)\nAUC = ", round(auc_val, 3)))
abline(a = 0, b = 1, lty = 2, col = "gray")
dev.off()
#


# 6. Single-Gene Correlation Analysis of RFXANK#


exp_vst <- assay(vst(dds))
cor_res <- apply(exp_vst, 1, function(x) {
  cor.test(x, exp_vst["RFXANK", ], method = "pearson")$estimate
})
cor_res <- data.frame(gene = names(cor_res), correlation = cor_res)
cor_res <- cor_res[order(-abs(cor_res$correlation)), ]

top_genes <- head(cor_res$gene, 30)
mat <- exp_vst[top_genes, ]
mat <- mat[order(cor_res[top_genes, "correlation"], decreasing = TRUE), ]
annotation <- data.frame(RFXANK = exp_vst["RFXANK", ])
```

```
rownames(annotation) <- colnames(mat)

p_corr_heatmap <- pheatmap(mat, scale = "row", show_colnames = FALSE,
                           annotation_col = annotation,
                           main = "Top 30 genes correlated with RFXANK",
                           clustering_method = "ward.D2")
ggsave("RFXANK_corr_heatmap.pdf", p_corr_heatmap, width = 8, height = 6)

#
```

# 7. GO/KEGG Enrichment Analysis of Differentially Expressed Genes

```
#

sig_genes <- sig_deg$gene
entrez_ids <- bitr(sig_genes, fromType = "SYMBOL", toType = "ENTREZID", OrgDb = org.Hs.eg.db)
background <- rownames(exp_vst)
bg_entrez <- bitr(background, fromType = "SYMBOL", toType = "ENTREZID", OrgDb = org.Hs.eg.db)

go_enrich <- enrichGO(gene = entrez_ids$ENTREZID,
                      universe = bg_entrez$ENTREZID,
                      OrgDb = org.Hs.eg.db,
                      ont = "BP",
                      pAdjustMethod = "BH",
                      pvalueCutoff = 0.05,
                      qvalueCutoff = 0.2,
                      readable = TRUE)
kegg_enrich <- enrichKEGG(gene = entrez_ids$ENTREZID,
                          organism = "hsa",
                          pvalueCutoff = 0.05,
                          qvalueCutoff = 0.2)

pdf("GO_enrichment.pdf", width = 10, height = 8)
dotplot(go_enrich, showCategory = 20) + ggtitle("GO Biological Process Enrichment")
dev.off()

pdf("KEGG_enrichment.pdf", width = 10, height = 6)
dotplot(kegg_enrich, showCategory = 20) + ggtitle("KEGG Pathway Enrichment")
dev.off()

write.csv(as.data.frame(go_enrich), "GO_enrichment_results.csv", row.names = FALSE)
```

```
write.csv(as.data.frame(kegg_enrich), "KEGG_enrichment_results.csv", row.names = FALSE)

#
```

# 8. GSEA Enrichment Analysis of Differentially Expressed Genes (c2.cp.all.v2022.1.Hs.symbols.gmt)

```
gmt_file <- "c2.cp.all.v2022.1.Hs.symbols.gmt"      #
gmt <- getGmt(gmt_file)
term2gene <- data.frame(
  term = rep(names(gmt), lengths(gmt)),
  gene = unlist(lapply(gmt, geneIds))
)

gene_list <- deg_df$log2FoldChange
names(gene_list) <- deg_df$gene
gene_list <- sort(gene_list[!is.na(gene_list)], decreasing = TRUE)

gsea_results <- GSEA(geneList = gene_list,
                     TERM2GENE = term2gene,
                     pvalueCutoff = 0.05,
                     pAdjustMethod = "BH",
                     seed = 123)

pdf("GSEA_c2_all.pdf", width = 8, height = 6)
dotplot(gsea_results, showCategory = 10, split = ".sign") + facet_grid(.~.sign)
dev.off()

write.csv(as.data.frame(gsea_results), "GSEA_c2_all_results.csv", row.names = FALSE)
```

# 9. Immune Infiltration Analysis of RFXANK with 24 Immune Cell Types (ssGSEA)

```
# CSV File (Two Columns: cell_type, gene)

immune_genes <- read.csv("immune_cell_genes.csv", stringsAsFactors = FALSE)
immune_list <- split(immune_genes$gene, immune_genes$cell_type)

exp_vst_mat <- as.matrix(exp_vst)
ssgsea_scores <- gsva(exp_vst_mat, immune_list, method = "ssgsea",
                      kcdf = "Gaussian", mx.diff = TRUE, verbose = FALSE)

immune_score_df <- data.frame(t(ssgsea_scores), RFXANK = exp_vst["RFXANK", ])
```

```
cor_immune <- apply(immune_score_df[, -ncol(immune_score_df)], 2, function(x) {
  cor.test(x, immune_score_df$RFXANK, method = "spearman")$estimate
})
p_immune <- apply(immune_score_df[, -ncol(immune_score_df)], 2, function(x) {
  cor.test(x, immune_score_df$RFXANK, method = "spearman")$p.value
})
immune_cor_df <- data.frame(cell_type = names(cor_immune),
                            correlation = cor_immune,
                            p.value = p_immune)
immune_cor_df <- immune_cor_df[order(-abs(immune_cor_df$correlation)), ]

write.csv(immune_cor_df, "RFXANK_immune_correlations.csv", row.names = FALSE)

# Plot Correlation Heatmap
pheatmap(immune_cor_df$correlation,
         main = "Correlation between RFXANK and Immune Cells",
         labels_row = immune_cor_df$cell_type,
         cluster_rows = TRUE,
         color = colorRampPalette(c("blue", "white", "red"))(100),
         filename = "RFXANK_immune_correlation.pdf", width = 6, height = 8)

# Plot Immune Infiltration Heatmap for All Samples

sample_order <- order(immune_score_df$RFXANK)
heatmap_data <- t(ssgsea_scores)[sample_order, ]
pheatmap(heatmap_data, scale = "column",
         annotation_col = data.frame(RFXANK =
immune_score_df$RFXANK[sample_order]),
         show_colnames = FALSE,
         main = "ssGSEA scores of 24 immune cell types",
         filename = "immune_infiltration_heatmap.pdf", width = 8, height = 6)
```

10 GEO Dataset Analysis

```
library(GEOquery)
library(limma)
library(dplyr)
library(ggplot2)
library(pheatmap)
library(reshape2)

setwd("~/GSE45267_analysis")
```

```
#   Data Download
gse <- getGEO("GSE45267", GSEMatrix = TRUE, AnnotGPL = TRUE)
expr <- exprs(gse[[1]])
anno <- fData(gse[[1]])
pheno <- pData(phenoData(gse[[1]]))

# Extract Group Information (Tumor vs Normal)

group <- factor(ifelse(grepl("tumor", pheno$source_name_ch1, ignore.case = TRUE), "Tumor",
                              ifelse(grepl("normal",   pheno$source_name_ch1,   ignore.case   =
TRUE), "Normal", NA)))
valid <- !is.na(group)
expr <- expr[, valid]
group <- group[valid]
pheno <- pheno[valid, ]

# Probe-to-Gene Symbol Conversion (Retain Probe with Maximum Expression for Each Gene)
symbols <- anno$`Gene symbol`
keep <- !is.na(symbols) & symbols != ""
expr <- expr[keep, ]
symbols <- symbols[keep]

expr_df <- as.data.frame(expr)
expr_df$probe <- rownames(expr_df)
expr_df$symbol <- symbols
expr_df$max_expr <- apply(expr_df[, 1:(ncol(expr_df)-2)], 1, max)
expr_df <- expr_df[order(expr_df$symbol, -expr_df$max_expr), ]
expr_df <- expr_df[!duplicated(expr_df$symbol), ]
rownames(expr_df) <- expr_df$symbol
expr_matrix <- as.matrix(expr_df[, 1:(ncol(expr_df)-2)])

# limma Differential Expression Analysis

design <- model.matrix(~ 0 + group)
colnames(design) <- levels(group)
contrast <- makeContrasts(Tumor - Normal, levels = design)
fit <- lmFit(expr_matrix, design)
fit2 <- contrasts.fit(fit, contrast)
fit2 <- eBayes(fit2)
deg <- topTable(fit2, number = Inf, adjust.method = "BH")
deg$gene <- rownames(deg)
```
